# Initiation of Post-Primary Tuberculosis of the Lungs: Exploring the Secret Role of Bone Marrow Derived Stem Cells

**DOI:** 10.3389/fimmu.2020.594572

**Published:** 2021-01-21

**Authors:** Lekhika Pathak, Bikul Das

**Affiliations:** ^1^ Department of Stem Cell and Infectious Diseases, KaviKrishna Laboratory, Guwahati Biotech Park, Indian Institute of Technology, Guwahati, India; ^2^ KaviKrishna Telemedicine Care, Sualkuchi, India; ^3^ Department of Stem Cell and Infection, Thoreau Laboratory for Global Health, M2D2, University of Massachusetts, Lowell, MA, United States

**Keywords:** post-primary tuberculosis of the lungs, dormancy, reactivation, stem cell niche, bone marrow derived stem cells, altruistic stem cells, *Mycobacterium tuberculosis*

## Abstract

*Mycobacterium tuberculosis* (*Mtb*), the causative organism of pulmonary tuberculosis (PTB) now infects more than half of the world population. The efficient transmission strategy of the pathogen includes first remaining dormant inside the infected host, next undergoing reactivation to cause post-primary tuberculosis of the lungs (PPTBL) and then transmit *via* aerosol to the community. In this review, we are exploring recent findings on the role of bone marrow (BM) stem cell niche in *Mtb* dormancy and reactivation that may underlie the mechanisms of PPTBL development. We suggest that pathogen’s interaction with the stem cell niche may be relevant in potential inflammation induced PPTBL reactivation, which need significant research attention for the future development of novel preventive and therapeutic strategies for PPTBL, especially in a post COVID-19 pandemic world. Finally, we put forward potential animal models to study the stem cell basis of *Mtb* dormancy and reactivation.

## Introduction

Pulmonary tuberculosis (PTB) is a major global health disease. Each year nearly 10 million new PTB cases are reported as estimated by the world health organization (1). Then, these infected cases spread the disease in the community *via* aerosol, thus the bacterial transmission is maintained (2). Noticeably, humans are the only host in the entire animal kingdom where the bacteria can complete its transmission cycle under natural conditions (3). Therefore, any attempt to develop an effective policy to eradicate this pathogen from humans needs an appreciation of how the pathogen exploits immunocompetent adults to maximize its transmission success.

*Mtb* enters into the human host *via* aerosol, initiates a primary infection in the lungs, which generate active TB lesions including caseating granuloma formation (4–7). A vigorous cell-mediated immune response leads to eventual calcification of the granuloma, and the infected person develops a robust, life-long immunity against primary TB (8, 9). However, after 10–30 years of dormancy or latency, active TB lesions reappear in the apical part of the lungs as post-primary tuberculosis of the lungs (PPTBL) (2, 10, 11). Importantly, these PPTBL infected adults exhibit vigorous cell-mediated immunity (CMI) against *Mtb* as confirmed by a positive tuberculin test (8, 10–13). As the PPTBL progresses, pulmonary tissues are filled with heterogeneous types of granulomas that include active cavities, as well as fibrotic, non-progressive, sterile granulomas (14). These non-progressive granulomas are a highly organized structure (14), where the dormant bacilli remain in a standoff with the immune cells (9, 15, 16). This highly organized non-progressive granuloma structure is unique to human TB infection and is not present in mouse models of PTB (17–19). The active granulomas expand into nearby bronchioles, allowing the bacteria to enter into the sputum. Then, the infected person spreads aerosols containing live *Mtb* into the community by the process of vigorous coughing. The bacteria enter into a new host, initiate primary TB infection in the lungs, undergo latency for years and initiate PPTBL. Thus, the initiation of PPTBL occurs in the lungs of an adult with latent TB infection (LTBI) (the time period between primary infection and the clinical manifestation of PPTBL). Hence the disease is named as post-primary TB of the lungs or PPTBL (8, 20) and only by causing PPTBL, *Mtb* maintains transmission in human (2, 21).

**Figure 1:**
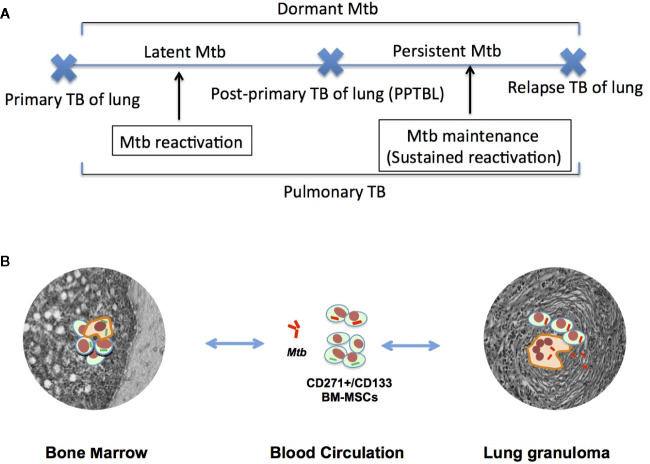
A hypothetical model of MSC mediated PPTBL development. **(A)** A schematic model of *Mtb* dormancy and reactivation showing the meaning of different terminologies used in the review article. **(B)** Hypothetical model showing the role of CD271+BM-MSC in PPTBL development. The model introduces a new self-renewing cell type, the CD271+BM-MSCs as a potential reservoir of dormant *Mtb* (d*Mtb*). Following primary TB infection, d*Mtb* hide in BM. In adults, inflammation in the lungs mobilizes BM-MSCs. The mobilization process facilitates homing of d*Mtb* harboring BM-MSCs into the lungs leading to PPTBL development [adapted from reference no. (22)].

The source of *Mtb* in the adult that initiate PPTBL is not yet clearly known, which limits our ability to target the transmission strategy of the pathogen in the community. From the perspective of *Mtb* transmission, the effective means for PPTBL development would be to hide in dormant state intracellular to a host cell type; which is immunosuppressed (21) and has self-renewal and migratory ability. Such a strategic approach would then permit the pathogen to migrate to apical part of the lungs to initiate a pneumonia-like exudative early phase of PPTBL. Previously, we proposed that the human adult stem cell niche in bone marrow (BM) might serve as a protective niche for dormant *Mtb* (22), and these cells would then migrate to lungs for PPTBL development (**Figure 1**). Surprisingly, many clinicians had already provided anecdotal evidence of finding *Mtb* in BM including our experience in Bhutan during 1995–1998 (23, 29) provide “bed to benchside” rationale to examine whether *Mtb* may hijack the BM-stem cell niche for its transmission strategy (22). However, skeptics will point out that hijacking of the stem cell niche will lead to widespread hematological and other stem cell-related disorders, often not seen in patients with PPTBL. We demonstrated that *Mtb* infects a rare population of human BM-stem cell to remain dormant (22). This may explain why hematological disorders are not widespread in PPTBL subjects. Following our initial findings, many laboratories not only reproduced our findings but also added important information about the *Mtb*/human BM-stem cell host-pathogen interaction (24–26). These findings raise human bone marrow derived stem cells as the site of *Mtb* latency (27). Recently, we have shown that stem cell altruism may be involved in *Mtb*-reactivation (28), and in the aerosol-induced immunity (29). These recent studies indicate the emerging role of BM stem cell niche in the pathogenesis and community transmission of *Mtb*.

The primary objective of this review is to discuss the significance of the adult stem cell niche as a protective site of TB dormancy and reactivation that allows the pathogen to initiate PPTBL in an immunocompetent adult and subsequently to transmit *Mtb* in the community. We speculate that during early phase of PPTBL initiation, *Mtb* harboring BM stem cells may mobilize, and home to lung for initiating PPTBL, and then exporting the bacteria to the community *via* sputum, thus completing the bacterial transmission cycle. Thus, in this review, we will put forward a new model of *Mtb* transmission in the community, and discuss the ways for the *Mtb* transmission cycle to be blocked by modulating the process of BM stem cell mobilization to lung. Furthermore, we put forward potential animal models to study the interaction between pathogen and stem cell niche so that critical mechanisms including putative stem cell niche defense mechanism can be studied and can be targeted to eliminate latent *Mtb* infection.

## The Enigmatic Source of Dormant *MTB* for the Initiation of PPTBL

A potential source of PPTBL is the exogenous re-infection with a different *Mtb* strain (30, 31), particularly in the geographical area of high TB incidence (31, 32). To explain the mechanism of PPTBL initiation in LTBI subjects, Medlar proposed the “allergic soil” hypothesis. Medlar suggested that the primary TB infection pre-conditions the apical part of the lungs for later migration of exogenous, re-infecting *Mtb* (5). However, the “allergic soil” hypothesis was ruled out in a guinea pig model of re-infection (33). Recent genome-based studies showed that endogenous or exogenous reactivation of *Mtb* could be evaluated by performing genotyping and whole genome sequencing (WGS) (34–36). However, it is not clear, how the bacteria from the exogenous re-infection would survive and initiate PPTBL in an immunocompetent adult host having vigorous cell mediated immunity against *Mtb* (21, 37–39). In this context, PPTBL initiation may rather be caused by endogenous *Mtb* strain that remained dormant in the human host after primary infection.

It is presumed that the endogenous source of the replicating *Mtb* in the PPTBL is the dormant *Mtb* hiding in the granulomas of lungs, and/or in the extra-granuloma sites in the lungs (3, 11, 12, 40–42). This presumption is supported by numerous animal models of *Mtb* infection, where, the dormant state is achieved as a result of *Mtb* interaction with the immune cells present in the granuloma (9, 19, 37, 43, 44) and the hypoxic microenvironment prevailing in the granuloma (45–49). However, the site of dormant *Mtb* in the lungs of LTBI subjects is not clearly known (4–6, 21, 39), although several probable sites have been proposed based on clinical findings (**Table 1**).

**Table 1:** Proposed endogenous sites for dormant *Mtb* in LTBI patients.

	Host cell type	Recovery of *Mtb* DNA	Recovery of viable *Mtb*	References
**Pulmonary**	Host cell free, sterile granuloma	Yes	No	(2, 4, 21, 38, 50, 51)
	^a^Lung tissue from subclinical cases	Yes	Yes	(52)
	Alveolar macrophages	Yes	No	(22, 37, 53, 54)
	Alveolar epithelial cells	Yes	No	(53)
**Extra-pulmonary**	Host cell free adipose tissue	Yes	No	(3, 21, 55)
	Adipcytes	Yes	No	(3, 21, 55)
	Macrophages	Yes	No	(21, 37, 55)
	Fibroblast	Yes	No	(21, 37, 55)
	^b^CD271+BM-MSCs	Yes	Yes	(22, 56, 57)
	^c^CD34+HSCs (Peripheral blood)	Yes	Yes	(22, 58)

**Figure 2:**
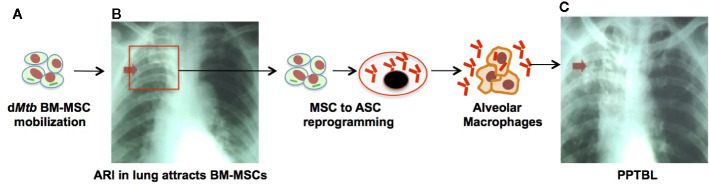
The emerging role of stem cell altruism in PPTBL development. **(A)** Inflammation such as acute respiratory tract infection (ARI) in lungs causes mobilization and homing of d*Mtb* harboring BM-MSCs to the lungs. **(B)** Inside the lungs, d*Mtb*-BM-MSCs self-renew and reprogram from MSCs to altruistic stem cells (ASCs) by the process of stem cell altruism (73, 112, 113). These ASCs undergo clonal proliferation and become permissible for intracellular replication of d*Mtb*. Replicating *Mtb* exit ASCs into extracellular space to infect alveolar macrophages. During this phase, patient exhibit subclinical exudative, focal pneumonia like phase of PPTBL as shown in chest X-ray of a patient with sub-clinical PPTBL (arrow). **(C)** A host immune response surrounds the infected alveolar macrophages, and eventually leads to granuloma and cavity formation, thus developing clinical PPTBL as shown in chest X-ray of a PPTBL patient (arrow).

We suggest that the finding imply early phase of dMtb mobilization to lungs and release of viable Mtb as explained in **Figure 2**.^a^Subclinical cases implies early pneumonia-like exudative early phase of PPTBL. ^b^CD271+BM-MSCs: CD271+bone marrow mesenchymal stem cells. ^c^CD34+HSCs: CD34+hematopoietic stem cells.

An obvious first site to look for the dormant *Mtb* would be the primary TB associated site, known as the “ghon complex”, a fibrotic granuloma located mostly in the lower lungs pleural area, and calcified hilar lymph nodes (2, 50). The granuloma becomes calcified and sterile 2–5 years after development of primary TB (2, 4, 21, 38, 50, 51). The site does not show any *Mtb* reactivation activity in PPTBL, which occurs 10–30 years after the primary TB (5, 20, 39). In fact, PPTBL occurs mostly in the apical part of the lungs (4, 21, 37, 50) instead in the primary TB infection site. Therefore, it is most unlikely that the primary granuloma is the site of *Mtb* dormancy and reactivation.

The second possible site would be the apical part of the lungs, the most common site of PPTBL. However, during the LTBI period, no dormant granulomas could be found at this site (4, 20, 34, 37, 50, 59). The third probable site is the normal lungs tissues. However, during LTB, no dormant granulomas containing viable *Mtb* could be found in the lungs (4, 20, 37, 42, 50, 59). Instead, human autopsy reports found a small amount of *Mtb*-DNA scattered intracellular and extracellular in the normal tissues of the lungs (21, 37, 53). Surprisingly, viable *Mtb* were isolated from normal lungs tissue of LTBI subjects in one report; however subsequent studies were not done to confirm whether the LTBI subjects were suffering from subclinical PPTBL infection (52). Some of the bacilli remain intracellular in alveolar macrophages (53). *Mtb* has been found to re-program macrophages to remain in a relatively non-replicative dormant state (9, 60); however, the viability of intracellular *Mtb* is poor (54), and so far, no direct isolation of viable *Mtb* from macrophages of LTBI subject has been reported (22, 37). Importantly, latent or dormant *Mtb* are highly immunogenic intracellular to macrophages as the bacteria fail to arrest phagosome maturation and are mostly processed for efficient antigen presentation to stimulate adaptive immunity (61). Thus, in LTBI subjects having vigorous T cell mediated immunity, it is highly unlikely that latent *Mtb* can persists intracellular to macrophages, as the pathogen will be killed by macrophage with the help of specific T cells mediated interferon gamma response (62).

The fourth potential site of dormancy is the putative alternate form of *Mtb* that includes endospores. However, such a phenotype of *Mtb* has not yet been identified, despite a century of research (37, 63).

The fifth potential source of PPTBL is the extra-pulmonary host cell types including adipocytes, fibroblasts and macrophages, where *Mtb*-DNA has been found (21, 37, 55). However, viable and dormant *Mtb* have not been recovered from these cell types. Furthermore, it is not clear how would *Mtb* migrate from these sites to the apical part of the lungs (59), because these cell types are differentiated and resident cells (22, 37).

## Bone Marrow (BM) Stem Cell Niche as the Site of Dormant *MTB*

BM is an important site of the adult stem cell niche, where hematopoietic, mesenchymal and endothelial stem cells reside in their quiescent state (64). The BM stem cell niche is immunopriviledged (65, 66). These stem cells have the potential to self-renew (64, 67–70) and expand in the hypoxia/oxidative stress microenvironment (71–75), prevalent in the area of inflammation. The hypoxic microenvironment of stem cell niche may favor *Mtb* dormancy (76, 77) and resistance against anti TB drugs (56, 57). All these reasons unite to make BM stem cells a potential niche for *Mtb* during LTBI (27). Interestingly, stem cell’s self-renewal property is utilized by other bacteria, including *Mycobacterium leprae* (78) and *Wolbachia* (79) for effective transmission. Thus, it is presumed that BM stem cells’ self-renewal property could be utilized by dormant *Mtb* for initiation and transmission of PPTBL.

To investigate BM-stem cells as the potential host cells for dormant *Mtb*, we first focused on developing an *in vitro* model of *Mtb* and human BM-stem cell host pathogen interaction. We used a serum free media to culture CD133+BM cells (22, 73). The CD133+BM cells are enriched in HSCs, endothelial stem cells (22, 58, 80, 81) and naïve CD271+MSCs (22, 73). We found that *Mtb* infect CD271+BM-MSCs and CD34+BM-HSCs but maintain a non-replicating dormant status mainly in the CD271+BM-MSCs for 2 weeks. These preliminary *in vitro* results helped us to focus on the CD271+ BM-MSC as a possible *Mtb* target for dormancy (22).

CD271+BM-MSC is a type of multipotent mesenchymal stem cell (66, 82–84) that is very rare, comprising only 0.0017–0.0201% of BM mononuclear cell compartment (85). The cell type has potent immunosuppressive activity (84) and resides in the immunoprivileged and hypoxic niche in BM (65, 66). The CD271+ BM-MSCs are highly heterogeneous, and co-express two hematopoietic stem cell markers, CD133 and CD34 (22, 66, 86). Several recent studies have demonstrated the potential *in vivo* self-renewal property of CD271+ BM-MSCs. In these studies, the CD271+ BM-MSCs were directly isolated from human BM by flow cytometry or magnetic sorting technique, and their multipotent differentiation capacity was confirmed (22, 66, 86). The *in vivo* BM niche of this cell type was also identified, where they remain in a quiescent state (66, 87), and then self-renew during mobilization in response to tissue damage (86).

We speculated that the stemness state (the stem cell state of undifferentiating and self-renewal) of stem cell could be one of the key mechanisms of *Mtb* dormancy in stem cells. The CD271+BM-MSCs, when grown *in vitro* in high serum media or with adipogenic agents, differentiate to mesenchymal stromal cells, including the loss of stemness markers CD271, CD133, ABCG2 and HIF-2alpha expression (22, 73). It also significantly reduces the viability of intracellular dormant *Mtb* (d*Mtb*) (22). These findings confirm the maintenance of the stemness state of stem cells is essential for *Mtb* dormancy.

To investigate if the CD271+BM-MSCs are the dormancy site for *Mtb* in PPTBL cases, we recruited patients through our KaviKrishna Telemedicine care (22, 29) located in NE India. These were successfully treated subjects for PPTBL, who donated 6–7 ml of BM for immunomagnetic separation of CD271+BM-MSCs. In the CD271+BM-MSCs from 9/11 subjects, *Mtb*-DNA was recovered, and two of these samples showed the presence of viable *Mtb* (22) (**Table 1**). Later, Tornack et al. also recovered *Mtb*-DNA from CD271+BM-MSCs of LTBI subjects (24). Thus, recovery of viable *Mtb* from CD271+BM-MSCs of PPTBL subjects successfully treated with anti-TB drugs indicates that BM stem cells may serve as a protective niche for *Mtb* against antibiotic treatment (22). Indeed, in a Cornell model of dormancy/reactivation, we recovered viable *Mtb* from the ABCG2+ expressing CD271+BM-MSCs despite prolonged anti-TB drug therapy (22, 56, 57). The MSCs expressing drug efflux pump, e.g. ABCG2 (22, 88, 89) might help intracellular *Mtb* to escape drug toxicity. Subsequently, we found that the *Mtb*-DNA harboring CD271+BM-MSCs of post-PPTBL subjects exhibited high expression of hypoxia inducible factor 1 alpha (HIF1alpha) and low expression of CD146 (a hypoxia down regulated cell surface marker) (56), suggesting that these *Mtb* infected BM-MSCs resided in the hypoxic niche of BM (56). Moreover, the hypoxic localization of these *Mtb*-harboring stem cells in the BM niche might make these *Mtb* unreachable by the current anti-TB therapy (56). Interestingly, hypoxia is known to induce dormancy in *Mtb* (45), and researchers found that *Mtb* intracellular to BM-stem cells of LTBI subjects express hypoxia induced dormancy genes DosR, hspX and c-lat (24). Overall, these findings suggest hypoxic niche of human BM-stem cells could be an important mechanism for *Mtb* dormancy.

Like human BM-MSCs, mouse BM-MSCs has also been found to contain dormant *Mtb* (22, 25). In a mouse model of *Mtb* dormancy, we recovered non-replicating dormant *Mtb* intracellular to CD271+BM-MSCs even after 6 months of primary TB infection. To confirm the long-term viability and re-infection capacity of these dormant *Mtb*, we performed the *in vivo* serial transplantation assay, where non-replicating *Mtb* harboring CD271+BM-MSCs from primary infected mice were injected into the secondary recipient mice. We showed that only a few of recovered dormant *Mtb*-m18b (~40) harboring CD271+BM-MSCs were enough to cause tubercular lesions in the lungs of secondary recipient mice (22), thus confirming the re-infection potential of dormant *Mtb* intracellular to CD271+BM-MSCs (22). Another study performed the expression of dormancy related genes in the *Mtb* recovered from CD45-Sca1+BM-MSCs; these cells were recovered from *Mtb* infected mice. The study found that recovered *Mtb* expresses dormancy related genes; thus confirming the dormancy status of the pathogen intracellular to Sca-1+BM-MSCs (25). However, *in vivo* transplantation assay was not performed to demonstrate the long-term viability of these dormant *Mtb* intracellular to Sca1+BM-MSCs.

The mechanism of *Mtb* dormancy intracellular to MSCs is now the subject of intense research. Using human and mouse mesenchymal stromal cells grown in the high serum media, several laboratories studied mechanisms of survival, adaptation and dormancy of *Mtb* intracellular to MSCs (24–26, 90). One of these studies found that *Mtb* remains in the lipid droplets inside the cytosolic fraction of MSCs induce lipid synthesis (25). Another study found that the cytosolic localization provides resistance capacity to *Mtb* against host cellular autophagy (91). Yet, another study found that virulent but not avirulent *Mtb* may reprogram BM-MSCs and remain viable inside them to escape cytotoxicity of antimicrobial peptide cathelicidin (90). A recent study found *Mtb* intracellular to BM-MSC exhibit increased expression of dormancy gene hspX with simultaneous increase in tolerance to anti TB therapy (26). Various studies showed that nitric oxide synthase 2 kills intracellular *Mtb* of MSCs by nitric oxide production (92–94), confirming the innate defense mechanism of MSC that may play important role in immune response against *Mtb* (95). In a model of human adipose tissue derived MSCs, virulent *Mtb* strain H37Rv exhibit a drug and inflammatory cytokine tolerant phenotype by modulating PGE2 signaling (96). These studies indicate the emerging significance of MSCs as a host cell for *Mtb* and also other pathogens (95, 97). Further studies using the *in vivo* naïve MSCs will be needed to determine the mechanism of *Mtb* dormancy intracellular to naïve human MSCs (73).

In addition to MSCs, HSCs may also be a potential niche for dormant *Mtb* (22, 24). Studies also showed that nitric oxide synthase 2 (Nos2) could play an important role in *Mtb* dormancy intracellular to HSCs (98). HSCs are the multipotent, self-renewing progenitor cells that reside in the BM niche in their quiescent state like MSCs (64, 65). In an invitro assay, we found that *Mtb* can infect CD34+HSCs (22). Furthermore, we found that HSCs of some of the previously treated PPTBL subjects contain dormant *Mtb* (22) (**Table 1**). Our finding was confirmed by Tornack et al. who recovered dormant *Mtb* in HSCs of LTBI human peripheral blood (24) (**Table 1**). When these human CD34+HSCs and mouse CD150+HSCs containing non-replicating *Mtb* were administered intratracheally to recipient immune-deficient mice, animals formed lesions in the lungs (24) suggesting that these dormant *Mtb* retained viability and re-infection capability. However, in mouse model of *Mtb* infection, HSCs has been found to resist internalization during acute infection (99).

Thus, we and others have identified MSCs and HSCs harboring dormant *Mtb*, and confirm their infectious and re-activating potential in a very limited number of subjects. However, a detailed study encompassing a larger group of individuals is required to test the hypothesis (22) that viable bacteria could be recovered from the BM-MSCs and HSCs of subjects with an early sub-clinical case of PPTBL.

## Bone Marrow Derived Stem Cells’ Potential Role in PPTBL: A Testable Hypothesis

The development of PPTBL occurs in adult LTBI subjects positive for IGRA (Interferon-Gamma Release Assays) or TST (tuberculin skin test). The clinical presentation of PPTBL occurs in seemingly healthy adults who had an episode of acute respiratory tract infection (ARI) for more than 2 weeks. Medlar tried to explain the development of PPTBL by the “allergic soil” hypothesis (4, 5). Accordingly, primary TB infection pre-condition the apical part of the lungs for migration and homing of bacteria (4, 5). Medlar hypothesis resembles the site-specific homing and niche to niche migration of MSCs (64, 67, 100–102) and HSCs (103–105). Accordingly, the primary TB infection may pre-condition the apical part of the lungs for migration and homing of *Mtb* infected MSCs and or HSCs in appropriate conditions. Such a possibility is gaining significance as our understanding of BM-stem cells migration and homing to distant organs is growing.

Recent advances in stem cell research suggest that BM-derived stem cells migrate to the area of inflammation, including lungs (106–109). The CD271+ BM-MSCs’ migration from BM niche into the circulation (104, 108, 109) following tissue damage/inflammation associated with acute myocardial infarction (86) and acute Ischemic Stroke (110) suggesting the mobilizations of these cells to the site of inflammation/injury. Additionally, BM-stem cells exhibit age-specific mobilization to specific tissues (104, 111). Moreover, CD271+ BM-MSCs are significantly mobilized in adult/elderly versus children (86). Interestingly, this is the age group of PPTBL development.

**Figure 3:**
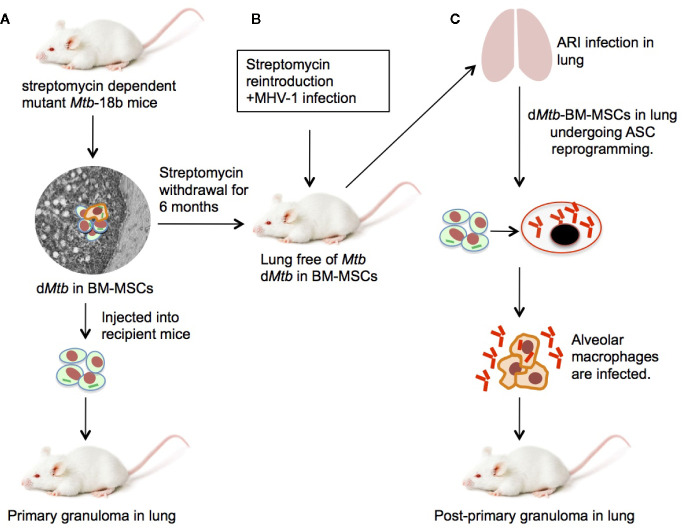
An experimental mouse model of stem cell mediated development of PPTBL. **(A)** Mouse model of streptomycin dependent mutant 18b-*Mtb* strain dormancy intracellular to BM-MSCs (22). **(B)** After 6 months of streptomycin starvation, streptomycin is re-introduced and mice are infected with MHV-1 intranasally to cause acute respiratory tract infection (ARI) inducing BM-MSC mobilization to lungs. **(C)** The d*Mtb* harboring BM-MSCs in MHV-1 infected lungs expands and reprogram to altruistic stem cells (ASCs; see text) that permit *Mtb* replication and exit to extracellular space. The reactivated *Mtb* then infect nearby alveolar macrophages, leading to PPTBL development.

Hence, based on these stem cells’ sites and age specific migration/homing potential, we have proposed a model of PPTBL development in an immunocompetent adult subject (22) (**Figure 1**). In this model, BM-stem cells harboring dormant *Mtb* may migrate to lungs in response to tissue inflammation likely due to ARI. These migratory stem cells will localize to the apical part of the lungs as per the site specific migration of CD271+ BM-MSCs and thus transfer *Mtb* to resident alveolar macrophages, as well as resident lung MSCs (**Figure 1**). As described in **Figure 2A**, periodic bouts of ARI may send signals from lungs to BM for stem cell mobilization as a part of BM-pulmonary niche to niche interaction. We hypothesize that d*Mtb* harboring MSCs will migrate to area of inflammation in the lung, and reprogram to the “enhanced stemness” a transient phenotype of stem cells characterized by ability to maintain stemness, as well as secret cytoprotective agents in the microenvironment of extreme oxidative stress/inflammation (72, 112). Stem cells that reprogram to “enhanced stemness” phenotype activate a HIF-2alpha stemness pathway, and exhibit altruistic behavior (112) i.e. sacrificing self-fitness to enhance group fitness during stress, and therefore, these transient stem cells can be termed as altruistic stem cells (ASCs) (73, 112) in contrast to competitive stem cells that eliminate weak neighbors during stress (114). ES cell derived ASCs exhibited intrinsic stemness (niche independent stemness i.e. autocrine regulation of stemness) having an altruistic component i.e. ability to modulate the niche to enhance group fitness in the microenvironment of hypoxia/oxidative stress (**Figure 3**) (112). Thus, ASCs exhibit niche modulatory or altruistic stemness in the microenvironment of hypoxia/oxidative stress, and therefore serve as niche defense mechanism (73). We suggest that pathogen may exploit stem cell altruism (112) to enhance their fitness in the hostile microenvironment of lung. Thus, intracellular d*Mtb* may facilitate ASC reprograming of MSCs in the ARI lung. The reprogrammed ASCs may then stimulate replicating of d*Mtb* and their subsequent release to neighboring MSCs and or macrophages. This process will lead to PPTBL development (**Figures 2B, C**). This model depicted in **Figure 2** can explain the clinical presentation of PPTBL in seemingly healthy adults who had an episode of ARI for more than 2 weeks before being diagnosed as a case of either sputum positive or negative PPTBL. The model can also explain the reactivation of dormant *Mtb* in immunocompetent adult despite having strong *Mtb* specific IFN-gamma producing CD4+/CD8+ T cells. We and others found that d*Mtb* harboring MSCs are cytoprotective (56) and can resist IFN-gamma mediated toxicity (96), which may protect the reactivating *Mtb* from immune onslaught.

The stem cell model of PPTBL may also help us to gain insight about sputum negative (*Mtb* negative for Acid Fast Bacilli stain and culture) PPTBL, where the lungs contain fibrotic, and non-progressive granulomas (14). These granulomas may contain mostly dead or deep-dormant *Mtb* that fails to stain for acid fast bacilli (AFB) (115), probably due to hypoxia/oxidative stress prevalent in the granuloma. These deep dormant *Mtb* are viable but non-culturable and require resuscitation factors (116). Clinical subjects of PPTBL harboring these granulomas may present in the clinic as sputum negative but Chest X-ray positive pulmonary TB (PTB) (117). Eventually, as the disease progress, the deep dormant bacteria in the granuloma in these patients may undergo resuscitation and therefore, patients turn into smear positive PTB. Thus, PPTBL progression and maintenance may involve a mechanism of resuscitating deep dormant bacteria. In this context, sputum of smear negative subjects contains viable but non-culturable (VBNC) *Mtb* (118); the source of this VBNC-*Mtb* may be lung granuloma as bacteria in sputum reflect active pulmonary lesions (119). Our preliminary study indicates that CD271+BM-MSCs may resuscitate VBNC deep dormant *Mtb* (29), suggesting the ability of stem cells to contribute to resuscitating dying granulomas. Recently, CD73+ cells containing *Mtb* antigen Ag85B has been detected in human lung granulomas (96), suggesting the potential presence of *Mtb* infected MSCs in active granuloma lesions of PTB, although further testing is required to confirm the MSC phenotype of the cells by direct sorting, as well as the viability of the intracellular *Mtb*. Thus, we propose that studying stem cell mediated resuscitation of deep dormant *Mtb* may contribute in understanding the pathogenesis of smear negative PTB.

## Developing Mouse Models of Stem Cell Mediated Initiation/Maintenance of PPTBL

Animal models are essential to gain insight about the role of BM stem cells in the endogenous reactivation of d*Mtb* leading to PPTBL. Guinea pigs, monkey, pigs, mice and rabbit are used to model TB infection. Among these animals, only in mice, the BM stem cell niche and resident lungs stem cells have been extensively studied. Hence, mice could be an appropriate animal model to study stem cells’ contribution to PPTBL initiation. Indeed, mouse model has been extensively used to study the immune equilibrium of lung granuloma (120), where MSC may contribute to favor *Mtb* growth (121). However, *Mtb* infected mice succumb to primary infection (22); therefore, PPTBL cannot be studied in this animal type. Additionally, Cornel model of dormancy is not considered as an appropriate model for PPTBL, as unlike in human, where immune cells induces dormancy, in the Cornell model, dormancy is achieved by treating animals with anti-TB drugs.

Nevertheless, we have used a streptomycin mutant strain of 18b (m18b) (122) infected mouse model to study PPTBL initiation. In this model, *Mtb*-m18b remains in viable and non-replicating state for 6 months intracellular to CD271+ BM-MSC population (22), mainly in the hypoxic niche (27). This mouse model is suitable to study stem cell mediated endogenous PPTBL, as the animal’s lungs become *Mtb* free after 6 months of streptomycin starvation; only a few *Mtb* in dormant state can be detected intracellular to lungs and BM CD271+MSCs (22), a situation similar to human spectrum of latent TB. Additionally, in this mouse model, we have fully characterized dormant *Mtb* harboring naïve MSCs in the lung and confirmed their stemness (self-renewal and differentiation). Therefore, in this mouse model, BM and lung MSC interaction can be evaluated during PPTBL development and maintenance. We have further improved this model by testing the idea of ARI mediated MSC to ASC reprograming and consequent *Mtb* reactivation as depicted in **Figures 2** to **3**. To investigate this possibility, dormant *Mtb*-m18b harboring mice were infected with an ARI causing corona virus, the MHV-1 (murine hepatitis virus strain-1) (123), to mimic oxidative/inflammatory stress in the lung. MHV-1 infection led to the ASC reprogramming of *Mtb* harboring CD271+MSCs. Importantly, ASC reprogramming led to *Mtb* reactivation/replication was reactivated in the lungs of 9/10 MHV-1 infected mice versus 1/10-control mice (28). Notably, the virus infected animal exhibited circulatory CD271+MSCs that contain *Mtb* (28) (**Figure 3**). We suggest that this MHV-1 infected mouse model of *Mtb*-m18b can be further improved as a putative mouse model of PPTBL. However, further studies are needed to confirm the usefulness of the mouse model to decipher the cellular and molecular mechanisms of *Mtb* dormancy in BM stem cells. Another approach would be to develop humanized mouse model that contains human immune system, so that dormant *Mtb* containing BM- stem cells of latent subjects can be injected to these animals. However, our last few years of attempt to develop this model were not successful as the humanized mouse model succumbs to *Mtb* infection within 2–3 months of infection.

## Future Direction

Numerous studies suggest that source of PPTBL initiation is the endogenous reactivation (34–36) although the source has not been clearly known. Recent studies suggest that human BM-stem cells may serve as a protective niche for dormant *Mtb* having reactivation potential (22, 24). Hence, future studies should be directed to confirm the role of BM-stem cells in endogenous reactivation of dormant *Mtb*. For clinical context, endogenous reactivation can be confirmed by collecting *Mtb* in stem cells of LTBI patients, and the *Mtb* obtained from sputum of the same patient following PPTBL, and then subjecting these *Mtb* to genotyping and WGS (34–36). This approach will confirm either the PPTBL is due to endogenous *Mtb* reactivation or exogenous *Mtb* strain. Such an approach may also be helpful to screen potential HSC transplantation donors. Reports showing *Mtb* infection after allogeneic HSC transplantation (124) suggest that it is necessary to confirm intracellular *Mtb* dormancy status of the donor to avoid risk of future d*Mtb* reactivation in the acceptor. In this context, we suggest that genotyping and WGS approaches may help to confirm the reactivation potential of dormant *Mtb* residing in donor’s BM-stem cells.

Future clinical studies should also be directed to examine if a dynamic interaction between the BM stem cell niche and lungs granulomas may contribute to PTB progression and drug-resistance. Stem cell model of *Mtb* dormancy/reactivation predicts that PPTBL mediated inflammation in the lungs will sustain the dynamic niche-to-niche interaction between BM-stem cells and lungs (**Figures 1** to **2**), thereby keeping granulomas alive. Pulmonary inflammation, especially in chronic obstructive pulmonary disease (COPD) and viral infection induced ARI may enhance the dynamic niche-to-niche interaction of BM stem cells and lungs niche thereby initiating and sustaining *Mtb* reactivation (28). Epidemiological data indicate that both smoking and COPD are two important risk factors for PPTBL initiation and relapse (125). Urban air pollution increases the incidence of COPD (126, 127), as well as pandemic such as COVID-19 related ARI (128). Importantly, both COPD and ARI may sustain and or aggravate the pulmonary inflammation. Hence, it is important to address the inflammatory aspect of PTB. Unfortunately, the inflammatory aspects of PTB are not well addressed clinically, and patients are left with inflammation, that may aid in COPD even after successful anti-TB therapy (125, 129, 130). In fact, millions of TB patients are suffering in their impoverished state in the developing world, mostly suffering from chronic lungs diseases including COPD related inflammation (126, 127) and ARI. Our two and half-decades of anecdotal experience in managing PPTBL subjects in Bhutan and India (23) through KaviKrishna telemedicine care (22, 29) (https://www.kavikrishnalab.org/ktc/) suggest that managing inflammation and improving the overall nutrition can reduce PPTBL development and relapse, consistent with published studies (131). Interestingly, a recent finding shows that anti-inflammatory drug celecoxib reduces the survival of *Mtb* intracellular to CD73+/Sca-1+ MSCs in the lung of INH treated mice with PTB (96) suggesting the potential benefit of managing inflammation to treat PPTBL. Whether such affordable intervention of anti-inflammatory agents reduces the dynamic interaction between stem cell niche and lungs granulomas is now under active investigation.

Further advance in the stem cell field is required to gain insight in to the mechanism of stem cell mediated *Mtb* dormancy and reactivation. It will be important to find out how the pathogen modulates stemness to maintain the immunosuppressive phenotype. Interestingly, *Mtb* infected adipose tissue derived MSCs has shown the activation of the autocrine pathway of PGE2 (96), which was earlier found to be involved in the stemness of MSCs. PGE2 may enhance the niche independent stemness of immunosuppressive MSCs (73), thus benefiting intracellular pathogen. BM stem cell niche may exert innate defense against pathogens by modulating the niche (73). Indeed, HSCs and endothelial progenitor cells modulate BM niche to prevent pathogen infection (132). We found that after intravenous injection of *Mtb* to mice, only a small fraction of BM-MSCs harbor the pathogen, suggesting that the stem cell may resist pathogen’s invasion. Indeed, *in vitro* studies found that intracellular *Mtb* are killed by autophagy and phagocytosis mechanism of MSCs (92). Also, *Mtb* infected MSCs secret nitric oxide (92) that kills the intracellular *Mtb* (93). Another *in vitro* study showed that rapamycin addition reduces the dormant *Mtb* load inside MSCs by inducing autophagy (25). These emerging data indicate a potential MSC mediated defense against *Mtb* invasion. Interestingly, pathogen including *Mycobacterium avium* may exhaust quiescence HSCs in BM niche by IFN-gamma mediated proliferation (133). Additionally, BCG or *Mtb*-H37Rv infection in mice BM causes HSC expansion (99, 134). These works suggest that pathogen may disturb the long-term self-renewal capacity of HSCs. In this context, we speculate that BM-stem cell niche has evolved niche defense mechanism to resist pathogen mediated HSC exhaustion. *Mtb*-H37Rv infection in BM causes the expansion of hypoxic MSCs (56), indicating the potential existence of a stem cell niche defense (73). However, in our Cornell model of dormancy study, *Mtb* seems to escape these mechanisms of stem cell niche defense to successfully reside inside BM-MSCs, while maintaining the long-term health of the animal (56), suggesting that HSCs were not exhausted. Thus, it appears that BM niche defense may have the ability to maintain HSC self-renewal despite pathogen invasion.

Understanding the mechanism of stem cell mediated defense against *Mtb* invasion will facilitate vaccine development against dormant *Mtb* (135). Our ongoing work on stem cell altruism (72, 73, 112) may help us to further gain insight about the putative ASC based stem cell niche defense and its role in *Mtb* dormancy and reactivation. First, we found that MHV-1 viral infection (a model of ARI) activates an innate ASC defense mechanism against the virus, and in the process, reactivation of d*Mtb* occur (**Figure 3**). Interestingly, MHV-1 infection serve as a mouse model of clinically relevant human infecting severe acute respiratory syndrome corona virus 1 (SARS-CoV-1) strain (123) and possibly SARS-CoV-2 mediated COVID-19. Thus, MHV-1 mouse model may be useful to study whether SARS-CoV-2 infection would reactivate dormant TB infection (28). Second, ASC reprograming mechanism may be of relevance in the resuscitation of deep dormant *Mtb* (29), which are VBNC (118). Importantly, we showed that VBNC obtained from *Mtb*-m18b strain present in the sputum of smear negative PTB subjects could be resuscitated by BM-MSCs and during the resuscitation process, MSCs reprogram to ASCs (29). Third, in a mouse model of *Mtb* infection, we found that VBNC harboring ASCs in the lung were identified and found to export extracellular vesicle (EV) into the broncho-alveolar (BAL) fluid. The EVs are rich in ESAT-6 antigen and therefore, may serve as natural aerosol based vaccine. Fourth, we also isolated such ESAT-6 rich EVs in the aerosol of subjects with smear-negative PTB. Notably, the EVs rich aerosols did not contain live *Mtb*. These findings indicate that aerosols of PTB subjects may transmit antigens into the community without spreading the pathogen, a potential natural vaccination process of herd immunity (29). We propose that studying ASC reprogramming may reveal an already existed natural immunity mechanism in the community against *Mtb*, which may further be utilized to develop an improved control program for TB or other pathogens (29).

We speculate that the work on *Mtb*/BM-stem cell host/pathogen interaction may also provide insight about the memory component of BM-stem cell niche defense. It is conceivable that stem cell niche has evolved sophisticated mechanism to defend their niche, including the retention of specific memory of a given pathogen. It has been known that innate defense mechanism is capable of specific memory (136) even in a thymic mice (137), and this type of innate immune memory is now known as trained immunity (138), and the mechanisms include the imprinting of pathogen specific epigenetic signature in innate immune cells and resident stem cells (138). Trained immunity has been largely characterized in BCG-vaccinated mouse model of memory macrophages (139) and memory NK cells (140). Importantly, in a stem cell model of trained immunity, BCG trained HSCs in BM may differentiate to monocyte and contribute to lung-alveolar macrophages defense against the invasion of virulent *Mtb* strain H37Rv (134). This indicates the role of trained immunity in the interaction between BM-stem cell niche and alveolar-macrophage compartment. Notably, virulent *Mtb* strain H37Rv modulates trained immunity of HSCs (99) and also modulate the hypoxic microenvironment of BM niche (56). Thus, virulent *Mtb* may have evolved mechanisms to evade trained immunity to remain dormant intracellular to MSCs or HSCs. Studying the mechanisms of *Mtb* mediated evasion of trained immunity to persist intracellular to HSC/MSCs may help to develop innovative vaccine strategies against tuberculosis. In this context, it will be interesting to study potential of *Mtb*-induced ASCs (28, 29) as a part of trained immunity.

The pathogenesis and the immune response in PPTBL is complex with multiple players often having double roles of pathology versus protection (141, 142). It is possible that stem cells may have double role: on one hand, stem cells may protect a community by spreading herd immunity (29), and on the other hand, stem cells may serve as a protective niche for dormant *Mtb*. To decipher the complex role of stem cells in pathogenesis and the immune response to PPTBL, future studies of BM-stem cell biology need to further address the i) stem cell niche based defense mechanism ii) stem cells’ niche to niche interaction between bone marrow and lungs iii) the role of stem cell niche defense in trained immunity.

## Discussion

In summary, emerging laboratory and clinical results now provide a conceptual framework of the potential role of adult stem cell niche in the dormant *Mtb* infection. Failure to eradicate TB, despite decades of TB control programs (143) may be due to dormant *Mtb* infection. The recent identification of CD271+ BM-MSCs, its localization in the hypoxic BM-niche (5, 66, 89), mobilization in response to tissue damage (88), and its ability to harbor dormant *Mtb* (9, 22) provide experimental support to this hypothesis. Importantly, our initial observations have been reproduced in many laboratories, which further strengthen our hypothesis. These findings suggest a deep evolutionary interaction between stem cells and *Mtb*, where the pathogen exploits self-renewal mechanism of stem cells to remain dormant and then induce PPTBL in healthy and immunocompetent hosts for their robust transmission in the human community. Advances in basic and translational biology research in stem cell and *Mtb* host/pathogen interaction is necessary in order to develop effective courses to eliminate this pathogen from human host.

## Author Contributions

BD has conceptualized, wrote and edited the manuscript. LP has wrote, edited the manuscript and created the figures and table. All authors contributed to the article and approved the submitted version.

## Funding

The work was supported by KaviKrishna Foundation, Sualkuchi, Assam (LP) (KKL/2018-4_MHV-1), Department of Biotechnology (DBT), Govt. of India (BD) (BT/PR22952/NER/95/572/2017) and KaviKrishna USA Foundation, Lincoln, MA (BD) (KUF/2019-ASC-BD).

## Conflict of Interest

The authors declare that the research was conducted in the absence of any commercial or financial relationships that could be construed as a potential conflict of interest.
